# Evaluation of a New Lightweight UAV-Borne Topo-Bathymetric LiDAR for Shallow Water Bathymetry and Object Detection

**DOI:** 10.3390/s22041379

**Published:** 2022-02-11

**Authors:** Dandi Wang, Shuai Xing, Yan He, Jiayong Yu, Qing Xu, Pengcheng Li

**Affiliations:** 1 The Institute of Geospatial Information, Strategic Support Force Information Engineering University, 62 Science Road, Zhengzhou 450001, China; wdd_93@163.com (D.W.); 13937169139@139.com (Q.X.); lpclqq@163.com (P.L.); 2 Science and Technology on Near-Surface Detection Laboratory, 160 Tonghui Road, Wuxi 214035, China; 3 Shanghai Institute of Optics and Fine Mechanics, Chinese Academy of Sciences, 390 Qinghe Road, Shanghai 201800, China; heyan@siom.ac.cn; 4 The School of Civil Engineering, Anhui Jianzhu University, 292 Ziyun Road, Hefei 230601, China; yujiayongskd@163.com

**Keywords:** LiDAR, bathymetry, unmanned aerial vehicles, object detection, coastal mapping, full-waveform

## Abstract

Airborne LiDAR bathymetry (ALB) has proven to be an effective technology for shallow water mapping. To collect data with a high point density, a lightweight dual-wavelength LiDAR system mounted on unmanned aerial vehicles (UAVs) was developed. This study presents and evaluates the system using the field data acquired from a flight test in Dazhou Island, China. In the precision and accuracy assessment, the local fitted planes extracted from the water surface points and the multibeam echosounder data are used as a reference for water surface and bottom measurements, respectively. For the bathymetric performance comparison, the study area is also measured with an ALB system installed on the manned aerial platform. The object detection capability of the system is examined with placed small cubes. Results show that the fitting precision of the water surface is 0.1227 m, and the absolute accuracy of the water bottom is 0.1268 m, both of which reach a decimeter level. Compared to the manned ALB system, the UAV-borne system provides higher resolution data with an average point density of 42 points/m^2^ and maximum detectable depth of 1.7–1.9 Secchi depths. In the point cloud of the water bottom, the existence of a 1-m target cube and the rough shape of a 2-m target cube are clearly observed at a depth of 12 m. The system shows great potential for flexible shallow water mapping and underwater object detection with promising results.

## 1. Introduction

With the development of laser technology, airborne LiDAR bathymetry (ALB) [1] has shown great potential in shallow water surveys in recent decades. ALB as an active remote sensing technology can measure the two-way transmission time from the sensor to the water surface and bottom by emitting a green laser pulse (532 nm) to penetrate the water. Thus, ALB systems can obtain the positions of water surface and bottom simultaneously by equipped inertial navigation and global navigation satellite systems. Compared with the multibeam echosounder (MBES) technology used extensively in bathymetry, ALB mainly has two advantages in shallow water mapping. One advantage is that the airborne platform is not constrained by the underwater terrain and is suitable for shallow water and coastal mapping [2]. The other advantage is that the swath width is determined by the flight altitude and scan angle rather than the water depth, so it can still maintain high efficiency in shallow water mapping [3]. Optical mapping sensors (RGB, multispectral cameras) also can be used for bathymetric surveying [4,5]. The application of Structure from Motion (SfM) technology [6] makes it possible to generate a 3D point cloud from overlapped aerial images, greatly improving the accuracy and spatial resolution of the bathymetric mapping. Although optical mapping sensors have advantages in low cost and high spectral resolution compared to ALB systems, it is difficult to retrieve water depths when the water bottom texture is unclear or invisible, and the depth accuracy is sensitive to the water surface conditions and water clarity [7].

Because the green laser requires sufficient pulse energy to penetrate the water, most ALB systems are designed for manned platforms, such as CZMIL, HawkEye 4X, and VQ-880-G. For eye safety considerations, the beam divergence of ALB systems is larger than topographic LiDAR and is normally greater than 1 mrad [8]. The flight altitude of the manned platform is commonly kept in the range of 300–500 m, so at a typical scan angle of 15°, a beam divergence of 1 mrad corresponds to a footprint diameter of 0.3–0.5 m [9]. The large beam divergence results in a large surface footprint size, which will influence the geometric precision.

Mounting the ALB system on unmanned aerial vehicles (UAVs) is an effective solution for collecting high-resolution measurements in some special cases, such as small island and reef mapping, underwater object detection, flood management, etc. The flying height of multi-copter UAVs can be set in the range of 20–50 m, reducing the footprint diameter to at least one-tenth that of manned aerial platforms. In addition, compared to manned aircraft, UAVs have the advantages of lower cost, easier operation, and freer airspace, allowing for more flexible and agile measurements [10,11]. However, due to the decrease in flight altitude, the swath width of UAVs is narrow (around 20 m), so they are only suitable for small areas survey.

In recent years, some ALB systems can be mounted on UAVs, called UAV-borne ALB systems. According to the sensor weight, the existing UAV-borne ALB systems can be divided into two categories. One is the lightweight system with a weight of about 5 kg, such as *RIEGL* BathyDepthFinder-1 (BDF-1) [12] and *ASTRALite* edge^TM^ [13]. The lightweight systems can be mounted on standard UAV platforms like the *DJI* Martice 600 Pro [14]. The benefit of the system is that it can operate at low altitudes with foot point diameters down to the centimeter level. However, laser energy is restricted due to the weight limitation, resulting in a weak bathymetric capability. Furthermore, the normal ALB systems commonly use circular or elliptical scanning patterns, while the lightweight systems use different ones. For example, the BDF-1 operates at a fixed 15° off-nadir, so it can only generate profile data [15]. Edge^TM^ uses a rectilinear scanning pattern resulting in a wide range of incident angles of the laser beam, which may affect the stability of the received signals [16]. The other type of UAV-borne ALB system is the compact system that weighs more than 10 kg and can be mounted on large-scale UAVs, such as *Fugro* RAMMS [17] and *RIEGL* VQ840-G [18]. The compact systems are miniaturized complete systems with comparable bathymetric performances and high point density. The maximum measured water depth can reach 3 Secchi depths (SD) [19], and the point density is approximately 20–50 points/m^2^ [20].

The existing UAV-borne ALB systems have been successfully applied to map inland rivers and lakes [20,21,22], showing the potential to offer better accuracy and higher spatial resolution data. In 2021, a new experimental lightweight UAV-borne ALB system, Mapper4000U, was developed by the Shanghai Institute of Optics and Fine Mechanics (SIOM), Chinese Academy of Science, and Strategic Support Force Information Engineering University. The weight of the sensor system is about 5 kg, so that it can be mounted on *DJI* Matrice 600 Pro. The system can simultaneously emit near-infrared (NIR, 1064 nm) and green (532 nm) laser pulses with an elliptical scanning pattern, ensuring efficient measurements and stable data acquisition. Equipped with the dual-wavelength laser, it is capable of classifying land and water signals with high accuracy [23,24], which is crucial for areas with blurred or irregular land-water boundaries. Another advantage of the dual-wavelength laser is that it can improve the accuracy of the water surface position when the water is turbid [25,26]. Recently, a flight test was conducted with the Mapper4000U in Dazhou Island, China. In this paper, the experimental results were presented, and compared to the MBES and manned ALB data acquired during the same period. The performances of the Mapper4000U, including the capability of bathymetry and object detection, were thoroughly evaluated.

The main contribution of this paper is providing a comparative analysis of the bathymetric performance of a new lightweight UAV-borne ALB system using the field data (UAV-borne ALB data, manned ALB data and MBES data) in a coastal area. The local plane fitting errors of the water surface points are analyzed to assess the relative accuracy of the water surface measurements of the UAV-borne ALB system. To estimate the bathymetric accuracy of the system, the water bottom measurements obtained by the system and MBES are directly compared based on the ellipsoid heights. The system is also compared with a manned ALB system in bathymetric performances, including the survey efficiency, point spatial distribution, measured depth range, and small object detection. Furthermore, the underwater object detection capability of the system is analyzed by manually placing ideal target cubes.

The rest of the paper is organized as follows. In Section 2, a detailed description of the Mapper 4000U is present. The study area, field datasets, and the methods for data processing and evaluation are introduced in Section 3. The experimental results are presented in Section 4 and discussed in Section 5. Finally, Section 6 summarizes this paper.

## 2. Mapper4000U

**Figure 1:**
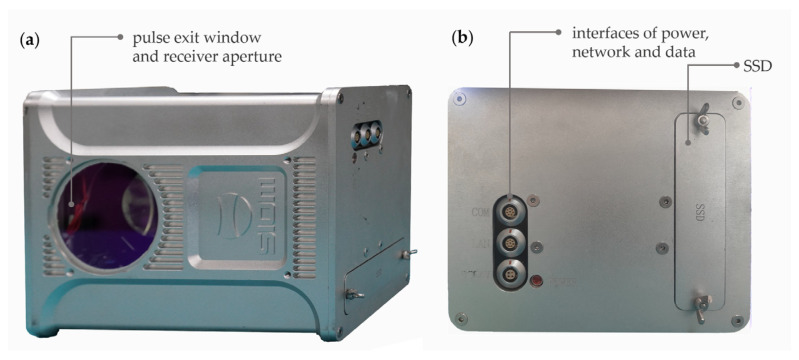
The appearance of the Mapper4000U; (**a**) the bottom of the system with the pulse exit window and receiver aperture for the near-infrared (NIR) and green laser; (**b**) the front side of the system with the interfaces and solid-state drive (SSD) socket.

The Mapper4000U is a lightweight and compact topo-bathymetric LiDAR system and is also a miniaturization of the *SIOM* Mapper5000 [27], which is designed for manned aerial platforms. The appearance of the sensor system is shown in Figure 1. There are two options to power it: using an external battery or a drone power supply. A solid-state drive (SSD) can be easily inserted to save data and unplugged to read data. The Mapper4000U can be connected to a digital camera for trigger control and can be integrated with a position and orientation system (POS) to build a comprehensive ALB system. As the sensor system only weighs 4.4 kg, it is flexible in selecting the POS and power supply solution to meet the payload restrictions of UAV platforms and the measurement requirements. The detailed system parameters are listed in Table 1.

The laser can emit both NIR and green pulses at a frequency of 4 kHz. A rotating scanner is used to create an elliptical scanning pattern with a scan angle of ±15° along-track and about ±12° cross-track. The beam divergence of the green laser is reduced from 1 mrad to 0.5 mrad because of the lower pulse energy. As a result, the device has a small footprint diameter (5 cm at an altitude of 50 m), ensuring that small features of the underwater terrain can be captured.

The receiver has two receiving channels, including an avalanche photodiode (APD) channel for the NIR laser pulse and a photomultiplier tube channel (PMT) for the green laser pulse. The PMT has a wide effective response range to avoid problems of signal saturation in extremely shallow waters [28] and ensure the ability of above 1.5 SD penetration. All the channels are sampled at a rate of 1G samples/s, i.e., the waveform data are recorded at an interval of 1 ns, corresponding to a slant distance of about 0.15 m in air and 0.11 m in water.

Compared to the other lightweight UAV-borne ALB systems, the Mapper4000U has better performances with a dual-wavelength laser and a rotating scanner. At the same time, the Mapper4000U still maintains the weight of a lightweight system and is more flexible than the existing compact UAV-borne ALB systems. In this paper, the UAV-borne ALB system is first presented and tested in a coastal area, and the bathymetric performance and object detection capability are evaluated.

## 3. Materials and Methods

### 3.1. Study Area

The study area Dazhou Island is located in the southeast of Wanning City, Hainan Province, China. The island is about 5 km off the coast of Wanning and consists of two ridges, a small northern ridge, and a large southern ridge, with a 500 m long beach in the middle. The study area is exactly in the sea between the two ridges. The water is clear, and the SD is in the range of 5–10 m. The underwater terrain is a continuous and gentle slope, and the water bottom is mostly covered by sand, which is suitable for assessing the bathymetric performance of the system.

**Figure 2:**
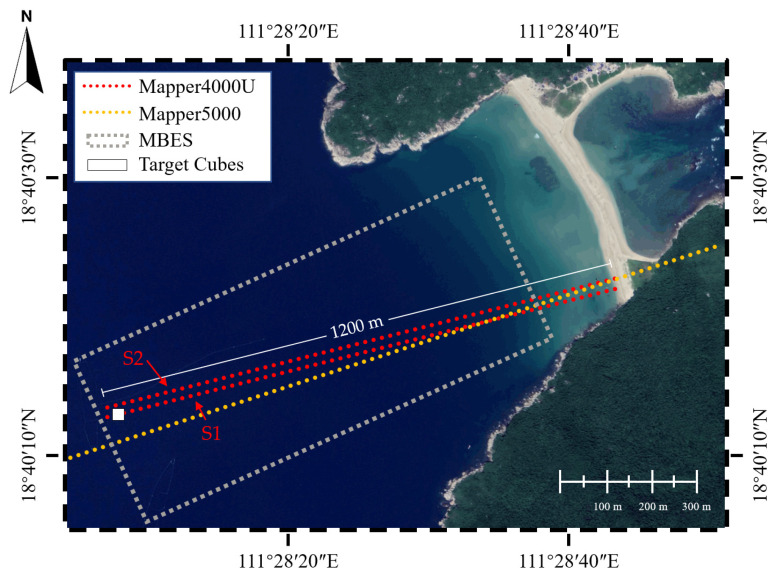
Study area. The red dotted lines denote the flight strips of the Mapper4000U, the yellow dotted line denotes the flight strip of the Mappper5000, the range of the multibeam echosounder (MBES) measurements is in the gray dashed box, and the location of the target cubes is denoted by the white box.

An 8-day survey for Dazhou Island was conducted from 25 September 2021 to 2 October 2021, as shown in Figure 2. The dates of data acquisition are shown in Table 2. The study was designed for a comprehensive performance evaluation of Mapper4000U, including a precision analysis, an accuracy assessment based on MBES reference data, a direct comparison between the manned (Mapper5000) and unmanned (Mapper4000U) ALB, and an object detection test. The field data contains two flight strips of the Mapper4000U, referred to as S1 and S2, one flight strip of the Mappper5000, and the MBES measurements.

### 3.2. Field Data

**Figure 3:**
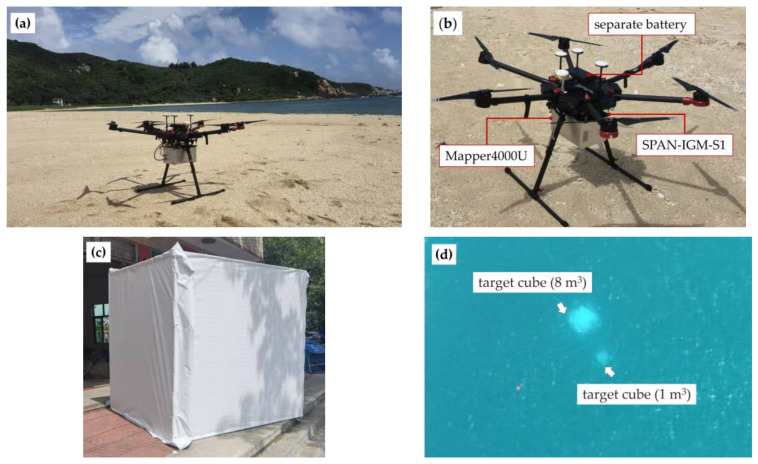
Photos of the Mapper4000U data acquisition and target placement; (**a**) Mapper4000U mounted on DJI Matrice 600 Pro; (**b**) detailed installation positions of the payloads; (**c**) a photo of the 2-m fabric cube; (**d**) an aerial image of the target cubes.

In this experiment, the Mapper4000U was powered by a separate battery and equipped with a POS (*NovAtel* SPAN-IGM-S1). The total weight of the system, including the sensor, POS, SSD, and battery, was 5.6 kg. The system was mounted on the *DJI* Matrice 600 Pro, as shown in Figure 3a,b. Using the standard batteries, a flight can last 10–15 min with approximately 30% power left. The flight altitude was kept at 50 m, and the swath width on the water surface of each strip was 21 m. The width of the overlap between S1 and S2 was 4 m, so the range of the measurement area was about 38 m × 1200 m. The specific data parameters are shown in Table 3.

In addition to the UAV survey, the experiment was carried out in the following steps:

1. For the accuracy assessment, the study area was also measured by a MBES (*Hydro-tech Marine* MS400), and a digital bathymetric model (DBM) [29] with high-resolution (0.2 m) was generated using the supporting software. The DBM was used as reference data of water bottom points in this experiment. The geographic coordinates of both the reference DBM and Mapper4000U survey points used the WGS84 ellipsoid and were projected into UTM zone 49 N.
2. For the bathymetric performance comparison, a Mapper5000 survey were performed a few days after the UAV survey. The flight altitude, speed, and swath width are much higher than that of the UAV, but the point density is sharply decreased. The data acquisition parameters are compared in Table 3. In data processing, a depth-adaptive waveform decomposition method was used for signal detection, and a post-processing software developed by the manufacturer was used for point cloud generation, including geo-calibration and refraction correction [27]. For comparison, the measured points were also transformed to the geographic coordinates (WGS84).
3. Small object detection capability. Two fabric targets, a 1-m white cube and a 2-m white cube, were placed in water one day before the UAV survey, and the location of the targets was measured at the same time. The cubes gradually sank to a depth of about 12 m, which was deeper than the Secchi depth.

### 3.3. Data Processing of the UAV-Borne ALB

The data processing of the Mapper4000U mainly consists of four steps:

1. POS data processing. The observations from the POS mounted on the platform and a temporary reference station were processed using Waypoint Inertial Explorer8.8 Software to estimate the flight trajectory.
2. Waveform data processing. The full waveforms were sampled and recorded by the receiver. To extract the signals, a fast and simplified processing method was applied to the received waveforms (see below for a detailed description).
3. System calibration. The system calibration was conducted in a nearby village. Six strips of Mapper4000U data were collected, and a number of control points were measured by RTK GNSS survey. Thus, the extrinsic error was corrected based on the planar calibration model [30].
4. Coordinates calculation. Based on the flight trajectory, the extrinsic parameters, and the refraction correction model [27], the detected signals were converted to the 3D point cloud in the WGS_1984_UTM_Zone_49N coordinate.

**Figure 4:**
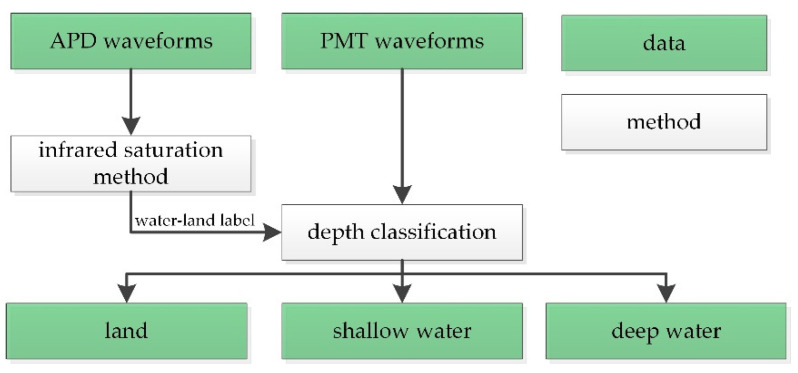
Workflow of the waveform classification. APD: avalanche photodiode; PMT: photomultiplier tube.

We simplified and modified the wave decomposition method proposed in [27] to fit the new system and improve the efficiency. Waveforms collected by Mapper4000U were classified into three categories, as shown in Figure 4, and then independently processed.

**Figure 5:**
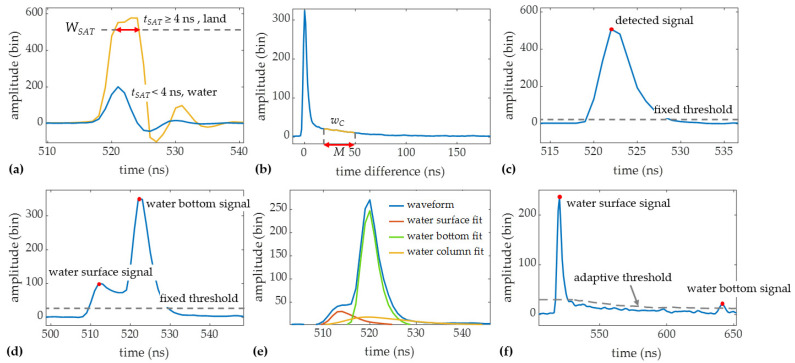
Schematic illustration of the waveform data processing; (**a**) water-land classification based on the infrared saturation method; (**b**) depth classification for water waveforms using the truncated water column scattering waveform (i.e., *w_C_*); (**c**) signal detection of the land waveform using the fixed threshold; (**d**) signal detection of the shallow water waveform using the fixed threshold; (**e**) empirical decomposition of the shallow water waveform; (**f**) signal detection of the deep-water waveform using the adaptive threshold.

Based on the infrared saturation method [31], waveforms were first classified into two groups, land and water, using APD waveforms. In the infrared saturation method, the saturation time of the APD waveforms is defined as the duration beyond the maximum effective value (saturated value) of the receiver. The saturation time *t_SAT_* can be expressed as follows:

(1) $$t_{SAT}=\sum_{t=0}^{N}w_{APD}\left(t\right)\geq W_{SAT},$$

where *w_APD_* is the APD waveform and *W_SAT_* denote the upper bound of the effective response range. The *W_SAT_* is the saturation value of the receiver’s output voltage, which can be easily determined from a waveform with a saturated signal. If *t_SAT_* is greater than or equal to the saturation threshold, the waveform will be labeled as “land”; otherwise, it will be labeled as “water” (see Figure 5a). The saturation threshold was set to 4 ns in this paper.

For “water” waveforms, further classification is needed because of the different characteristics of shallow and deep water waveforms. In the depth classification method [27], the similarity between the received waveforms and the water column scattering is measured, as shown in Figure 5b. The similarity *S* can be calculated as:

(2) S=min{R(t)},

(3) $$R\left(t\right)=\frac{1}{M}\sum_{m=1}^{M}\left[w_{C}\left(m\tau \right)-w_{PMT}\left(m\tau +t\right)\right],$$

where *w_PMT_* denotes the PMT waveform, *w_C_* is the truncated water column scattering waveform extracted from *w_PMT_*, *M* is the length of *w_C_*, and *τ* is the sampling interval. As the shape of the water column scattering changes little in a small survey area, the deep-water waveform will have a high similarity, while the shallow water waveform, where the water column scattering is mainly covered by the surface and bottom returns, will have a low similarity. Based on the value of *S*, the “water” waveforms were classified into two parts, “shallow water” and “deep water”.

PMT waveforms in the three categories were processed as follows:

- Land waveforms processing. Considering the gentle coastal terrain, only the moving-average algorithm [32] and a signal detection method with a fixed maximum threshold [33] are performed on the land waveforms, as shown in Figure 5c.
- Shallow water waveforms processing. Shallow water waveforms are first processed using the same methods as the land waveforms for signal detection. If the number of detected signals is greater than or equal to 2, the first signal will be recorded as the water surface signal, and the last signal will be recorded as the water bottom signal, as shown in Figure 5d. If only one signal can be detected, which occurs when the water depth is extremely shallow, the waveform will be decomposed based on an empirical model [27] to extract the water surface and bottom signal, as shown in Figure 5e.
- Deep-water waveforms processing. Denoising is the key to deep water waveforms processing, while the existing waveform filtering methods cannot appropriately deal with the high-intensity noise in the water column scattering. Thus, the fixed threshold in the signal detection method is replaced by a depth-adaptive threshold derived from the truncated water column scattering waveform [27], which greatly reduces the effect of noise in the water column scattering. The intensities of the detected signal are subtracted from the depth-adaptive threshold, and the two signals with the highest strength are selected as the water surface and bottom signal, as shown in Figure 5f.

### 3.4. Methods for the Evaluation

The performance of the Mapper4000U were evaluated in four aspects, and the evaluation methods in [20] were partially used here.

1. Precision: Analysis of the relative accuracy of the measurements. The water surface points of each strip were searched in 1 m × 1 m grids based on the planimetric coordinates. In each grid, the points were fitted to a plane, and the distance from the point to the plane was calculated to estimate the ranging precision. In addition, the DBMs of the water bottom were generated via the moving least squares interpolation and compared in the overlapping area of the two strips to evaluate the consistency of the data.
2. Accuracy: Assessment of the UAV system’s bathymetric accuracy. As the bathymetric LiDAR and MBES only measure instantaneous depths, the depth measurements cannot be directly compared. Therefore, the accuracy was evaluated by comparing the ellipsoid heights of the measured water bottom points with the reference values derived from the DBM generated by MBES measurements in the same planimetric coordinates.
3. Bathymetric performance: A comparison of the Mapper4000U and Mapper5000 for bathymetry (including point density, maximum depth penetration, and object detection capability). The point distribution and average density were both considered, and the profiles of the water bottom point clouds obtained from the UAV-borne system and manned platform system were compared. The maximum detected depths of the systems were estimated with the Secchi depth as reference. The capability of small object detection was examined in shallow and deep water.
4. Object detection capability: The target cube points were extracted from the water bottom point cloud of Mapper4000U and were fitted and projected to planes. To assess the detection accuracy, the distances from the points to the fitted plane and the shape of the projected points were statistically analyzed.

## 4. Results

### 4.1. Precision

The precision of the measured water surface reflects the ranging accuracy of the system and affects the refraction correction results, which indirectly influences the accuracy of the water bottom points. Since the water surface height is instantaneous, the reference data should be measured simultaneously. Although the APD channel can obtain a more accurate water surface, the instability of the signals makes it impossible to generate a high-density reference point cloud [34]. Based on the spatial coherence of the water surface [35], we obtained the references of the water surface height by local plane fitting. The error of the water surface height (i.e., *δ*_S_) is estimated according to the height difference, which is defined as:

(4) $$\delta_{S}=h_{S}-{h^{\prime }}_{S},$$

where *h*_S_ is the ellipsoid height of the measured water surface points and *h′*_S_ is the ellipsoid height of the fitted plane in the same planimetric coordinate.

**Figure 6:**
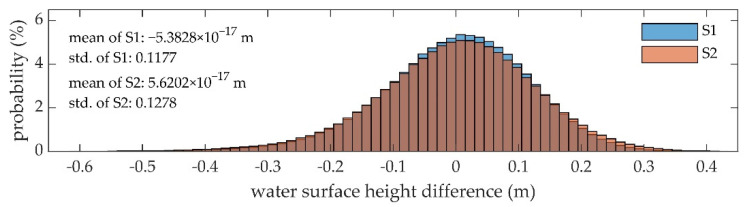
Probability distributions of the water surface height differences between measured points and fitted planes of S1 (blue) and S2 (orange); std.: standard deviation.

The histograms of the height deviations of the two strips, S1 and S2, are shown in Figure 6. The error distributions of S1 and S2 are approximately the same, which follow the Gaussian distribution and are centered around the zero value. In contrast, the distribution of S1 is slightly concentrated.

The errors of the water surface height are presented numerically in Table 4. The deviation between the mean water surface heights of S1 and S2 is 5.5 mm. The mean water level elevation (MWLE) in the study area at the time of the UAV survey is about −7.9478 m. The root mean square error (RMSE) of S1 is slightly lower than that of S2. The overall RMSE of the water surface height is below 0.13 m and 98% of the height errors are within ±0.3 m.

To assess the consistency of the measurements, the height differences of the water bottom points in the overlapping area of S1 and S2 were analyzed. First, the water bottom points of the two strips were converted from the UTM coordinate to a relative coordinate system with the *x*-axis parallel to the direction of the strips. Then two digital elevation models with 1 m resolution for S1 and S2 (DBM1 and DBM2) were created along-track using moving least squares interpolation, respectively. The DBM1 was subtracted from the DBM2 within the overlapping range to obtain the height differences of the water bottom (dDBM).

**Figure 7:**
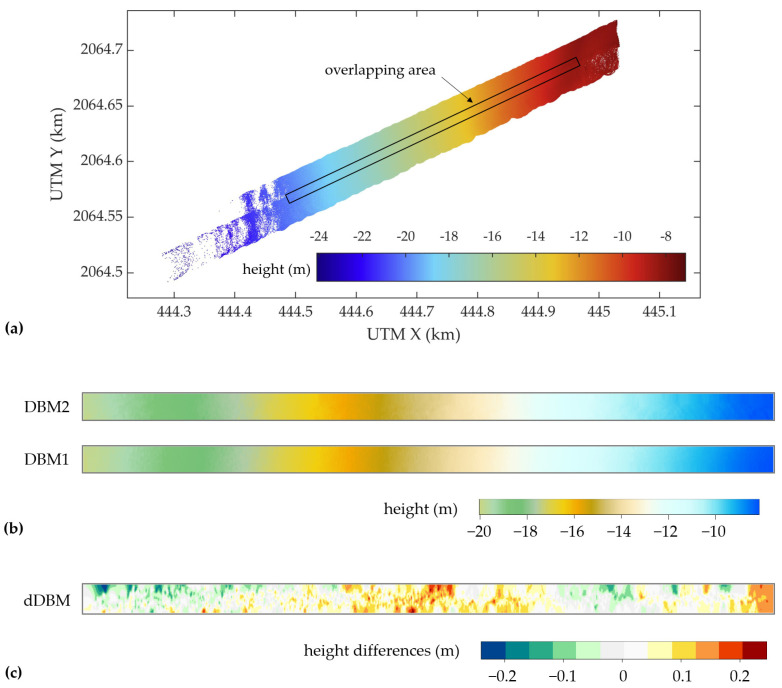
Comparison of water bottom height in the overlapping area; (**a**) color-coded map of the water bottom height of S1 and S2 with the overlapping area marked in the black box; (**b**) DBM1 and DBM2 in the overlapping area; (**c**) the height differences between the two strips in the overlapping area; DBM: digital bathymetric model.

Figure 7 shows the water bottom measurements of S1 and S2 with the location of the overlapping area and DBM1, DBM2, and dDBM in the overlapping range. By comparing DBM1 and DBM2, it can be observed that the elevations of the water bottom points in the same planimetric coordinates are almost equal, showing high consistency between the measurement results of the two strips. In dDBM, the height deviations are mainly within ±0.2 m. The height differences are regionally distributed, with negative values concentrated in the upper left and positive values in the middle and rightmost.

**Figure 8:**
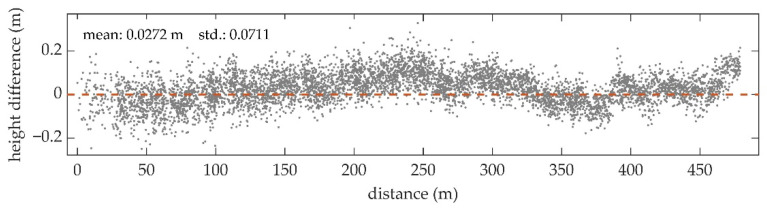
Distribution of the water bottom height differences along the direction of the strips, where the horizontal coordinates are the distances from the grids to the leftmost grid in the strip direction, and the vertical coordinates are the elevation value of dDBM.

In Figure 8, a profile of the dDBM in the vertical direction is plotted. The DBM1 is averagely 2.72 cm higher than the DBM2, and the dispersion of the height difference is 7.11 cm. According to the DBM1 and DBM2 shown in Figure 7, the water depth decreases gradually from left to right, while the height differences of the water bottom rise first, then fall, and finally rise again. Thus, the trend of the height differences is not consistent with that of the water depths.

### 4.2. Accuracy

The accuracy of the measured water bottom ellipsoid heights *h*_B_ directly represents the bathymetric accuracy of the system. In this experiment, the ellipsoid height references *h′*_B_ were obtained from the DBM of MBES. The error of the water bottom height (i.e., *δ*_B_) can be expressed as

(5) $$\delta_{B}=h_{B}-{h^{\prime }}_{B}.$$

**Figure 9:**
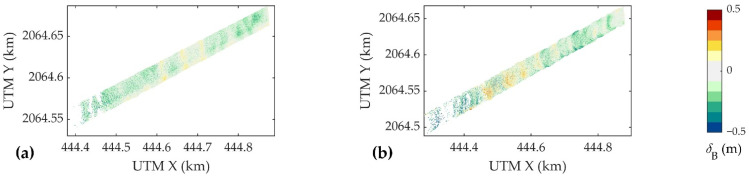
Color-coded maps of the water bottom height errors of (**a**) S1 and (**b**) S2.

Figure 9 shows the spatial distribution of *δ*_B_, where the right side is the water near the beach (i.e., the water depth gradually increases from right to left). The majority of the errors are negative, indicating that the ellipsoid height of the water bottom point is generally lower than the reference value. The water bottom points with large errors are mainly distributed in deep waters, especially in S2, where some water bottom points with the *δ*_B_ close to ±0.5 m exist. Comparing the error distributions of S1 and S2, it can be seen that the accuracy of S1 is higher, which is consistent with the precision assessment of the water surface height in Section 4.1.

Table 5 summarizes the statistical analysis of the water bottom height. According to the range of the height and the MWLE (−7.9478 m) estimated in Section 4.1, the range of the detectable depth of Mapper4000U is 0–16 m. The SD in the survey area was around 8.33 m which was estimated by the aerial images simultaneously acquired by *DJI* Phantom 4 pro. Based on the position of the deepest underwater object that could be visually observed from the images, the SD in the survey area was approximated by the corresponding water depth acquired from the manned ALB system. Thus, the maximum detectable depth corresponds to 1.7–1.9 SD. The overall RMSE of the water bottom height is 0.1268 m, which is larger than that of the water surface height. Different from the water surface points, the accuracy of the water bottom points in S1 is distinct from that in S2, with a difference of 3.45 cm. The mean of *δ*_B_ for both S1 and S2 is negative, resulting in an overestimation of depth which is also found in the measurements of VQ840-G [20]. For the requirement of the vertical accuracy (within ±0.3 m), more than 96% of the water bottom points are qualified.

**Figure 10:**
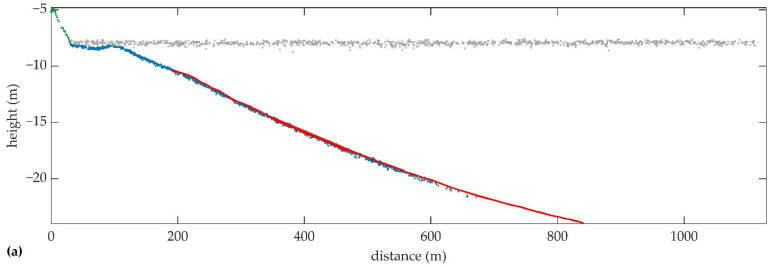

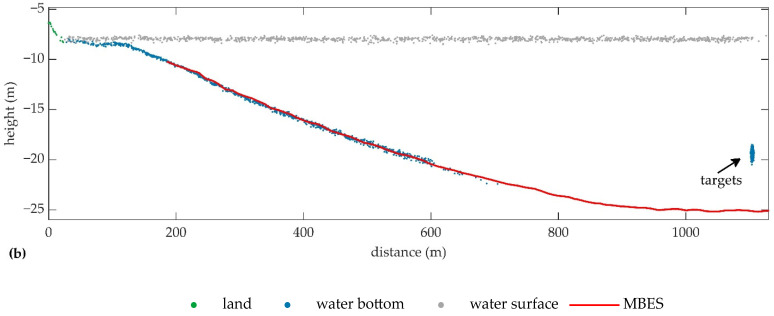
Profiles of the 3D point clouds of (**a**) S1 and (**b**) S2 colored by classification, and the DBM obtained by MBES, where the horizontal coordinates are the distances to the point with maximum X-coordinate in the UTM coordinate system.

The profiles of the point clouds of S1 and S2 along the center of the strip are shown in Figure 10a,b, respectively, where all the target points are plotted as water bottom on the profile of S2 regardless of the distances to the profile. Most of the water bottom points are below the references, which is also observed in Figure 9 and Table 5. When the height is below −20 m, the error of the water bottom point increases significantly. The dispersion of the water bottom points in S2 is greater, which is consistent with the statistical results in Table 5. Both in S1 and S2, the land points and water bottom points at the land-water interface are well connected, and the topography is continuous with slight height differences. The depth of the targets is about 12 m, which exceeds the SD in the survey area.

### 4.3. Bathymetric Performance

**Figure 11:**
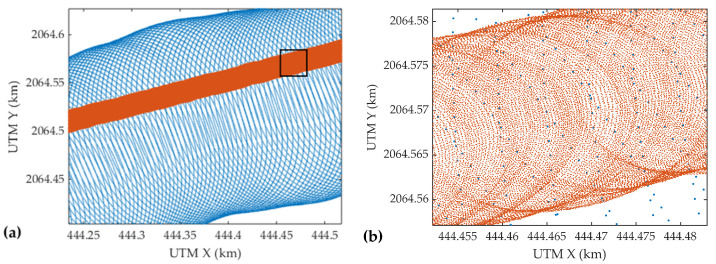
Distributions of water surface points acquired by the Mapper4000U (red dots) and the Mapper5000 (blue dots) on the XY plane; (**a**) the overall distribution; (**b**) the detailed distribution (zoom in the black box).

The bathymetric performances of the UAV-borne ALB system (Mapper4000U) and the manned ALB system (Mapper5000) were compared in this experiment. Figure 11 presents the distributions of water surface points, where the red dots denote the point cloud of S1, and the blue dots are the point cloud of the selected strip of Mapper5000 (for location, see Figure 2). Owing to the elliptical scanning pattern, the point clouds of both Mapper4000U and Mapper5000 are unevenly distributed, with points sparse in the middle and dense on the sides. There are two scanning lines at one position, and the trajectories of the forward and backward scanning are crossed. The point density of Mapper4000U is significantly higher than that of Mapper5000, while the swath is narrower. According to the specifications shown in Table 3, the average point density of Mapper4000U is 42 points/m^2^, which is 110 times that of Mapper5000, but the swath is only 21 m, which is only one-tenth that of Mapper5000.

**Figure 12:**
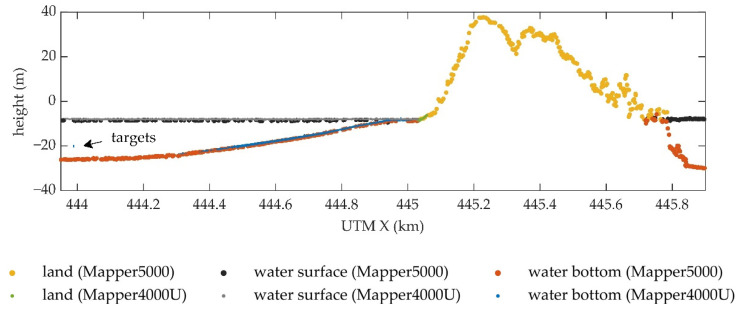
Profiles of the 3D point clouds obtained by the Mapper4000U and Mapper5000.

Figure 12 shows the cross-section of the point clouds of S2 and Mapper5000 strip along the track of S2. Both systems can acquire land, water surface, and water bottom points simultaneously. The MWLE varies slightly due to the different dates of data acquisition. The heights of the land and water bottom points obtained from the two platforms are very close. Most of the water bottom points obtained from the UAV are above those obtained from the manned aircraft. Since the overestimation of water depth in UAV data was found in Section 4.2, this problem is still present and even more serious in the manned platform data. Based on the heights of the water surface points, the MWLE at the time of the Mapper5000 survey can be estimated, and the result is −8.36 m. Since the minimum height of the water bottom points of Mapper5000 in the study area is −34.33 m, the maximum detectable depth of the system is 25.97 m, corresponding to 3 SD.

**Figure 13:**
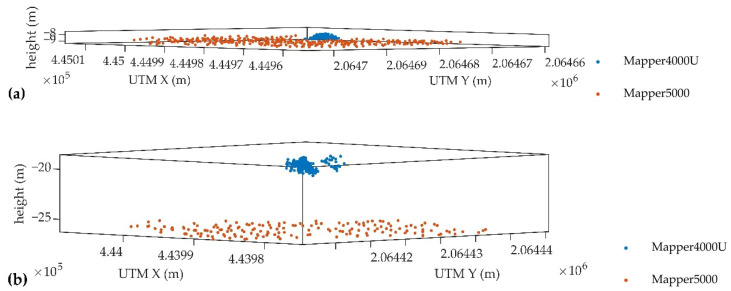
Perspective view of 3D point clouds of (**a**) a 3 m width circular mound and (**b**) the placed targets.

Although the ALB system mounted on the manned platform can measure deeper waters compared to the UAV system, it is unable to keep the detailed topographic features due to the low point density and large footprint size. For example, a circular mound and the placed targets can be clearly identified in the point cloud of the Mapper4000U. The shape of the circular mound is reserved, while the targets suffer a severe shape deformation. However, the circular mound and the placed targets are failed to be found in the point cloud of the Mapper5000, as shown in Figure 13.

### 4.4. Object Detection Capability

In the point cloud of S2, two clusters of target points were detected, and their corresponding targets were judged based on the volumes of the clustered points. Two planes can be clearly observed in the point cloud of the 2-m cube, so the points are fitted separately, namely P1 and P2. Since the shape of the point cloud of the 1-m cube is hard to be identified, all the points are fitted to a plane, namely P3.

**Figure 14:**
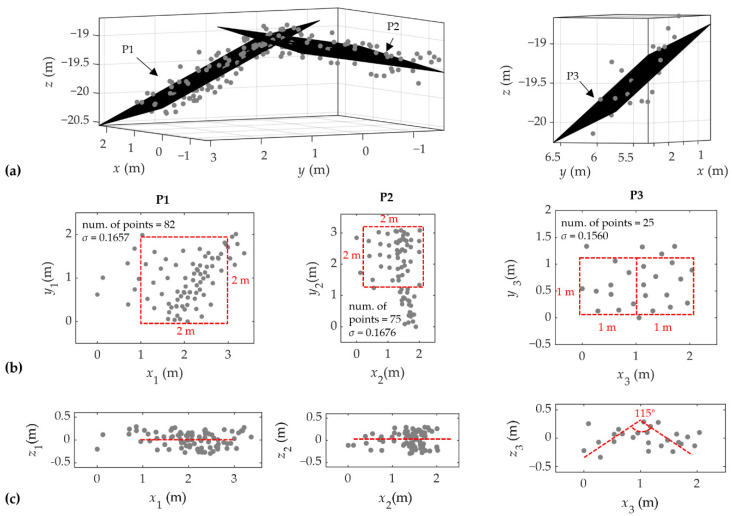
Results of the target points fitting; (**a**) perspective view of 3D point clouds of the 2-m target cube and 1-m target cube with the fitted planes P1–P3; (**b**) target points projected to P1–P3 with the dispersion of the point-to-plane distance (i.e., *σ*); (**c**) vertical section of the target points.

The plane fitting results of the target points are presented in Figure 14. For the convenience of presentation, the 3D fitting results are plotted under a relative coordinate system, i.e., the origin of the UTM is translated on the XY plane, and 2D fitting results are plotted under the relative coordinate system established for each fitted plane individually. For the 2-m target cube, the angle between the normal vectors of P1 and P2 is 36°, the number of points in each plane is approximately 80, and the fitting error is less than 0.17 m. From the projection of the points in the fitted plane, we can see that the coverage area of the points is larger than the standard 2-m square, and the deformation is obvious. From the profile, the error of the target points is within ±0.5 m, but the target points are more divergent than the water bottom points. For the 1-m target cube, the number of the point cloud is only 25. From the projection in the fitted plane, the shape of the points is similar to a combination of two 1-m squares, and the area is close to 1 m × 2 m. However, from the direction perpendicular to the plane, it is not easily identified as a composition of two faces. If the point clouds on the left and right sides are fitted with lines, respectively, the angle between the lines is about 115°.

## 5. Discussion

### 5.1. Environmental Effects on Water Surface Detection

For the precision of the water surface, the error distributions of S1 and S2 are similar, as shown in Figure 6, indicating that the error is independent of the measurement location and flight direction. In addition to the systematic errors, the influence of environmental factors cannot be ignored. For example, when the water surface signal is weak, the detected peak is a mixed peak of the water surface signal and the water column backscattering, so the position of the water surface signal cannot be accurately estimated, which is called the water surface uncertainty of the green laser [1,26]. As a result, the detected water surface will be lower than the actual water surface. As the slope and roughness of the water surface are random, the intensity of the water surface signal cannot be predicted, introducing a random error in the water surface signal detection. Furthermore, the sampling rate of the receiver is 1 GHz (c.f. Table 1); that is, the interval between two adjacent samples in the waveform is 1 ns, which corresponds to a distance of 0.15 m on the slant path in the air. Since the water surface signal position acquired by the signal detection method can only be an integer, an approximation error is introduced to the water surface, reaching a theoretical maximum of 0.075 m. Although waveform decomposition can eliminate the effect of the water surface uncertainty and obtain accurate results, it is quite time-consuming and is not suitable for emergency usage. One of the feasible solutions is to increase the sampling rate of the receiver, and the other is to optimize the current processing method to fast estimate the accurate position of the water surface signal.

### 5.2. Consistency between the Adjent Strips

Because of the narrow swath of the UAV-borne system, the matching error of the strips needs to be considered. In the evaluation of the strip consistency, the height difference of the water bottom between the two unmatched strips is generally within ±0.2 m. Therefore, the results can be directly used for DBM generation without strip adjustment if the accuracy of the products is not strictly required. Regional distribution of the height difference is observed in Figure 8, showing that there may be a small deviation between the two strips. It is expected to produce a more accurate DBM by correcting this error through the strip adjustment methods such as the iterative closest point (ICP) algorithm.

### 5.3. Impact of In-Water Path Calculation on Water Bottom

In ALB, the water bottom positions are derived from the water surface measurements and the in-water path of the laser pulse. Unlike the error distribution of the water surface points, the error distribution of the water bottom points is not uniform, and the error rises as the water depth increases. The calculation of the in-water path consists of three elements, the starting point (i.e., the water surface point), the path direction, and the propagation velocity. The accuracy of the water surface measurements has been discussed in Section 5.1. The water surface, as the interface between air and water, will affect the refraction correction. If the detected water bottom signal is accurate, a low water surface position induced by water surface uncertainty will have a downward vertical shift impact on the water bottom point, which may be responsible for the overestimation of water depths (c.f. Table 5).

However, the random error caused by water surface uncertainty cannot explain the problem that the water bottom error grows with the water depth. The error induced by the refraction direction accumulates with depth, which may be the main reason for the large errors in deep waters. Waves can affect the slope of the water surface and thus change the direction of the in-water path, which severely influences the UAV-borne ALB system with a small footprint size [36,37]. In [35], a 3D water surface profile model was built to predict the water surface slope, which is expected to solve this problem.

Another factor that affects the in-water path is the propagation velocity. The refractive index of the water determines the in-water propagation direction and velocity of the laser pulse and is usually fixed at 1.33. Recent studies have found that the fixed refractive index of 1.33 is inappropriate [20]. Using a group velocity instead of the fixed refractive index can reduce the range-dependent bias of the water depth [38]. Therefore, the calculation of the in-water path has a significant effect on water bottom, especially in deep waters, which need to be carefully corrected.

### 5.4. Bathymetric Performance Comparison

From the examined bathymetric performances of the manned and UAV-borne ALB system, it is found that manned ALB systems have the advantages of fast flight speed, extensive coverage, high efficiency, strong power energy, and a wide range of detectable depth. The drawbacks are the low point density and large footprint size, making it difficult to measure the complex features of the terrain accurately. Furthermore, the placed targets are invisible in the point cloud of the Mapper5000. One possibility is that the interval between the measurement and target placement lasts a few days (c.f. Table 2), and the target shifted during this period. Another possibility is that the points are too sparse, and the target points are mistakenly removed as noise during the point filtering process. According to the average point density, a 2-m square corresponds to about 1.5 points. If the targets do not locate at the edge of the strip, where the point density is relatively high, they will be difficult to detect. The advantages of the UAV-borne ALB system are the extremely high point density and small footprint size, which ensure sufficient detected points and accurate measurements, besides the maximum detectable depth of 2 SD can meet the demands of most shallow water surveys. The circular mound in shallow water and the placed targets in deep water all can be clearly observed in the point cloud (see Figure 13). In addition, the UAV platform is easy to operate and can finish the measurement of two 21 m × 1200 m strips in one flight. The flight duration is only 10 min including take-off, landing, and calibration. For a nearshore area with a range of 500 m × 1200 m, at least 30 flight strips need to be measured with 20% strip overlap, which takes 150 min in total. Although the UAV platform is less efficient than the manned aerial platform according to the speed and swath width, UAVs, especially multi-copter UAVs, which can vertical take-off and landing, have a few requirements for environmental conditions. As a result, the flight preparation time is greatly reduced, and the measurement efficiency is indirectly improved.

The bathymetric performances of the UAV-borne ALB system and MBES can also be compared in Figure 10. The advantages of MBES are high accuracy, high point density, and the ability to measure extremely deep waters. However, the minimum detectable depth of MBES is limited by the navigable area of the ship-borne platform. ALB can obtain land, water surface, and water bottom point clouds simultaneously. With the equipped NIR laser, the land and water points can be precisely distinguished. The excellent shallow-water measurement capability of ALB can complement the data of MBES. For example, in this experiment, the minimum depth measured by MBES is 2.31 m, while the maximum depth measured by the UAV-borne ALB system is 16 m. There is enough overlapping area that allows one to merge the measurements of MBES and ALB.

In summary, ALB is good at shallow water surveys, where the manned ALB is suitable for regular large area survey tasks, and the UAV-borne ALB is applicable for detailed surveys in small areas, besides MBES can help ALB to complete measurements in deep water. However, both ALB and MBES lack spectral information, limiting their further applications. The combination of ALB, MBES, and optical mapping sensors has been extensively researched and widely applied for inland water surveying [39,40,41] and coastal shallow water mapping [42,43]. Considering that the lightweight UAV-borne ALB system allows flexible installation, the integration of the system and optical cameras can be mounted on various UAV platforms. The fusion of the high spatial resolution LiDAR point cloud and high spectral resolution images in future work will provide a promising solution to coastal management.

### 5.5. Evaluation of the Object Detection Capability

To assess the object detection capability, two fabric cubes were placed in the water. Although both the 1-m cube and 2-m cube are identified in the point cloud, deformations and irregular shapes are also obvious. Two faces can be observed in the 2-m cube, but the angle between them is not equal to 90°. For the 1-m cube, it is impossible to identify the original shape from the point cloud. However, the underwater topography can be clearly observed in shallow water, such as a 3 m width circular mound (see Figure 13). Therefore, the accumulation errors induced by the in-water path are possibly responsible for the deformations of the target cubes. Because the bathymetric accuracy of the system gradually reduces with the increase in water depth (c.f. Figure 9), the target points at a depth of 12 m are highly affected. Small footprints of the system improve the resolution of the measurement and magnify the influence of in-water path errors. Thus, the calibration of the in-water path is crucial for object detection, especially for targets in deep water. In the absence of wave correction and propagation velocity correction, the Mapper4000U can determine the existence of a 1-m target cube and the rough shape of a 2-m target cube at a depth of 12 m.

It is worth mentioning that the reflectance of the object is also crucial for detection because it is directly related to the received power of the system [44]. A similar target cube is used to examine the object detection capability in [45], from which it is known that the reflectance of a white metal cube is 40–45% while the seafloor reflectance is around 10–20%. As the target cubes used in this experiment are ideal, natural objects will be more difficult to detect due to the low reflectance.

## 6. Conclusions

In this study, a new lightweight UAV-borne ALB system is presented and evaluated. The system weighs less than 5 kg and can be easily mounted on a multi-copter UAV platform like *DJI* Matrice 600 Pro. The system equipped with a dual-wavelength laser can flexibly measure shallow waters in small areas at a pulse repetition frequency of 4 kHz and a scanning speed of 15 lines/s.

To assess the system performance, we conducted a flight test at Dazhou Island, China, and acquired field data of two strips. From the experimental results, the main conclusions are as follows.

1. The system can simultaneously acquire land, water surface, and water bottom point clouds with a maximum detectable depth of 1.7–1.9 SD.
2. The accuracy of the system is evaluated from two aspects, water surface, and bottom. The RMSE of the water surface and bottom heights are 0.1227 m and 0.1268 m, respectively. The detection of the surface signal may be influenced by the water column backscattering, which may also be one of the reasons for the overestimation of water depths. Affected by the calculation error of the in-water path of the laser pulse, errors of water bottom points are dependent on water depths.
3. Compared to the manned ALB system, this system is lighter and more flexible and can preserve more detailed topographic features with 110 times the point density of the Mapper5000.
4. For object detection, the system can successfully detect white fabric cubes at a depth of 12 m (beyond 1 SD). The presence of the 1-m target cube and the general shape of the 2-m target cube can be observed in the point cloud. However, shape deformations of the targets also can be observed because of the depth-dependent errors, and the possibility of an object being detected is affected by its reflectance.

The experimental results have demonstrated the measurement accuracy, bathymetric performance, and object detection capability of the Mapper4000U. However, there are still some aspects that need to be further researched. For the hardware, the sampling rate of the receiver and the repetition frequency of the laser are expected to be increased, so the accuracy of signal detection and point density can be improved. In terms of the data processing software, the correction of the water column backscattering effects and the calibration of the in-water path need to be added to the data processing procedure to enhance the detectability of small objects in deep waters.

## Figures and Tables

**Table 1:** Mapper4000U specifications.

Pulse Repetition Frequency	Pulse Energy	Scan Rate	Size	Weight
4 kHz	12 μJ@1064 nm 24 μJ@532 nm	15 lines/s	235 mm × 184 mm × 148 mm	4.4 kg

**Table 2:** Data acquisition dates.

Mapper4000U	Mapper5000	MBES	Target Placement
26 September	2 October	29 September	25 September

**Table 3:** Data acquisition parameters of the Mapper4000U and Mapper5000.

System	Altitude	Speed	Swath Width	Point Density	Flight Duration ^1^
Mapper4000U	50 m	5 m/s	21 m	42 points/m^2^	225 s
Mapper5000	375 m	65 m/s	201 m	0.38 points/m^2^	22 s

^1^ Only the flight time of each strip was counted.

**Table 4:** Statistics of water surface ellipsoid height including mean, RMSE, and percentage of the error within ±0.3 m.

Strip	Mean of Height [m]	RMSE [m]	|*δ*_S_| < 0.3 m [%]
S1	−7.9452	0.1177	98.49
S2	−7.9507	0.1278	97.49
Sum	−7.9478	0.1227	98.01

**Table 5:** Statistics of water bottom ellipsoid height including maximum (max.), minimum (min.), RMSE, mean of the error and percentage of the error within ± 0.3 m.

Strip	Max. of Height [m]	Min. of Height [m]	RMSE [m]	Mean of *δ*_B_ [m]	|*δ*_B_| < 0.3 m [%]
S1	−7.9500	−22.1639	0.1075	−0.0520	98.48
S2	−8.0665	−24.1038	0.1420	−0.0705	93.89
Sum	−7.9500	−24.1038	0.1268	−0.0615	96.11
